# Phenotypic Alterations of CD8+ and CD8− CD56+ NK Cell Subsets in Moderate and Severe COVID‐19 Disease

**DOI:** 10.1155/jimr/8647361

**Published:** 2026-09-12

**Authors:** Matyas Meggyes, David U. Nagy, Ildiko Toth, Livia Mezosi, David Sipos, Agnes Peterfalvi, Laszlo Szereday

**Affiliations:** ^1^ Department of Medical Microbiology, Medical School, University of Pecs, Pecs 7624, Hungary, pte.hu; ^2^ Institute for Ecology, Evolution and Diversity, Faculty of Biological Sciences, Goethe University Frankfurt, Frankfurt am Main, Germany, goethe-university-frankfurt.de; ^3^ Department of Anesthesiology and Intensive Therapy, Medical School, University of Pecs, Pecs 7624, Hungary, pte.hu; ^4^ 1st Department of Medicine, Division of Infectious Diseases, Medical School, University of Pecs, Pecs 7624, Hungary, pte.hu; ^5^ Department of Laboratory Medicine, Medical School, University of Pecs, Pecs 7624, Hungary, pte.hu

**Keywords:** CD107a, CD8, COVID-19, immune checkpoints, NK cells, SARS-Cov-2

## Abstract

The COVID‐19 pandemic has created a global health challenge. Severe cases are associated with immune system dysfunction, which can lead to uncontrolled inflammation. There are no published data on the specific roles of natural killer (NK)‐cell subsets and immune checkpoint (IC) molecules in disease severity. Thirty‐five patients diagnosed with COVID‐19 and 14 healthy controls were involved in the study. From peripheral blood, CD56dim and CD56bright cell subsets were analyzed by flow cytometry for the expression of IC molecules (T‐cell immunoglobulin and ITIM domain [TIGIT], CD226, and PD‐1), activation markers (CD69), and cytotoxic potential (CD107a degranulation, granzymes, and perforin content). In COVID‐19 patients, the proportion of CD8− CD56dim cells was significantly higher than the CD8+ subset. Inhibitory receptors TIGIT and PD‐1 exhibited significantly higher relative expression in CD8+ CD56dim cells compared to their CD8− counterparts across infected groups, an effect particularly pronounced in deceased patients. Conversely, activating CD226 expression was reduced in the CD8− CD56dim subset only in severe cases. Functional assays revealed significantly elevated CD107a and CD69 expression in CD56dim cells of patients versus controls. Notably, CD8− CD56bright cells from deceased patients demonstrated enhanced CD107a expression and elevated perforin content, suggesting a shift toward hyperactivation. The preferential upregulation of inhibitory checkpoint molecules on the highly active CD8+ subset may reflect a compensatory response to immune activation, whereas the enhanced activation of CD8− NK‐cell subsets was associated with disease severity and mortality. Similarly, the heightened cytotoxic profile of CD8− CD56bright cells observed in fatal cases may reflect immune dysregulation associated with severe COVID‐19.

## 1. Introduction

The current COVID‐19 pandemic represents an unprecedented global health crisis. While most infections are mild or asymptomatic, some patients progress to severe, life‐threatening illness [1, 2]. Disease severity and mortality are influenced by a combination of factors, including demographic and socioeconomic variables, preexisting conditions such as obesity and diabetes and gender [3]. Key features of severe infection include lymphopenia and aberrant T‐cell activity, which together cause immune dysregulation and uncontrolled inflammation [4]. A serious complication of COVID‐19 is the cytokine storm, which results from an exaggerated surge of inflammatory mediators, specifically proinflammatory interleukins such as IL‐1β, IL‐6, and TNFα [5, 6]. While cytokines are vital for infection clearance, an overactive immune response can lead to tissue destruction, multiorgan failure, and mortality [5]. It is worth noting; however, that the occurrence of a cytokine storm does not uniformly reflect the disease severity.

Natural killer (NK) cells are now recognized for their dual functionality, effectively bridging the innate and adaptive immune systems, a role that is crucial for their efficacy against malignant transformation and viral pathogens [7, 8]. Their defining characteristic is an inherent, nonsensitized capacity to identify and lyse abnormal cellular targets. The maintenance of systemic homeostasis and key processes such as gestation and immune modulation relies heavily on the complex interplay between NK cells and their microenvironment. Peripheral NK cells represent 5%–10% of peripheral lymphocytes. The principal cytotoxic subset is the CD56dim population (>90%), characterized by low CD56 expression [9]. Conversely, the less numerous CD56bright subset (10%) exhibits high CD56 expression and is primarily composed of regulatory cells [10]. The surface expression of CD8 on a subset of NK cells suggests a possible lineage link or functional parallel with cytotoxic T lymphocytes. CD8+ NK cells are detected in a variety of pathological states, including infection, autoimmunity, and oncology [11–13]. The precise functional significance of the CD8 molecule on NK cells remains incompletely understood and is the subject of ongoing detailed investigation.

Immune checkpoint (IC) molecules are vital regulators that balance pro‐ and anti‐inflammatory immune responses. Their receptor–ligand interactions transmit costimulatory or coinhibitory signals that determine the cell’s function toward inflammation or tolerance. While IC molecules, particularly inhibitors, are extensively studied in tumor immunology [14, 15], data concerning their role in viral infection are limited and often contradictory. Previous publications reported that the PD‐1/PD‐L1 axis can mediate immune responses and contribute to COVID‐19 pathogenesis; however, these studies did not include a detailed analysis of NK‐cell subpopulations [16–18].

T‐cell immunoglobulin and ITIM domain (TIGIT) is an inhibitory IC receptor found on activated immune cells like CD4+ T cells, Treg cells, NKT cells, and NK cells [19–21]. TIGIT is known to suppress CD8+ T‐cell effector functions, decrease NK‐cell cytotoxicity, and lower TCR expression [22–24]. CD226 (DNAM‐1) is a competitive costimulatory molecule of TIGIT and is expressed mainly on the surface of NK cells, CD8+ T cells, CD4+ T cells, and monocytes [25]. TIGIT and CD226 compete to bind to the common ligands CD155 and CD112. Crucially, the interaction between CD155 and CD226 mediates activating signals [26], which are essential for proper NK and CD8+ T‐cell responses and promote anti‐tumor activity [27, 28]. At the same time, the CD155/TIGIT connection transmits inhibitory signals to the TIGIT‐expressing effector cells [23].

This study’s primary objective is to investigate immune differences between patients with moderate and severe COVID‐19, focusing on CD8‐positive and CD8‐negative NK cell subpopulations. In our previous work [29, 30], we examined in detail the relationship between immune cell subpopulations and the aforementioned IC pathways in patients with moderate and severe COVID‐19. However, there is no available data about the association of CD8+ and CD8− NK cell subpopulations in the clinical outcome of patients with COVID‐19.

## 2. Materials and Methods

### 2.1. Clinical Study Design (Demographics and Basic Characteristics of Severe and Moderate COVID‐19 Patients)

This clinical study involved a cohort of 35 COVID‐19 patients admitted to the University of Pécs, Hungary, between April 13 and December 7, 2021, during the Delta variant’s predominant third wave of the pandemic. None of the enrolled patients had documented bacterial, viral, or fungal coinfections at the time of hospital admission. The cohort was divided by disease severity. Eighteen patients with moderate severity were admitted to the infectious disease unit. Seventeen patients with severe disease requiring intensive care were admitted to the intensive care unit. Notably, the severe group had a mortality rate of 64%. Inclusion was based on the Cappanera et al. [31] quick COVID‐19 cytokine storm score, which considers five key laboratory values: lymphocyte count < 1000 cells/mm^3^, D‐dimer > 1000 ng/mL, LDH > 300 IU/L), ferritin > 500 ng/mL, and CRP > 10 mg/dL. SARS‐CoV‐2 infection was confirmed upon admission using RT‐PCR tests (LightMix Modular E‐ and N‐gene kits on a Cobas Z 480 PCR platform). Disease severity and prognosis were assessed using the sequential organ failure assessment (SOFA) score, simplified acute physiology score (SAPS), and a computed tomography (CT) score of lung involvement. Detailed cohort information is in Table 1.

**Table 1:** Demographic characteristics of the involved patients.

Variables	Healthy controls (*n* = 14)	Patients with moderate infection (*n* = 18)	Patients with severe infection (*n* = 17)	*p*‐Value
Age, mean, years (±SD)	44 ± 13	52.2 ± 11	66 ± 14	<0.05 vs. HC <0.05 vs. IDU
Females/males	6/8	6/12	6/11	NS
Obesity (BMI, median) (min., max.)	NA	27.78(19.11, 45.2)	29.4 (21.48, 71.78)	NS
Comorbidities				
Hypertension (*n*, %)	0	6 (33.3%)	14 (82.4%)	<0.01 vs. IDU
Heart disease (*n*, %)	0	0	3 (17.6%)	NS
Chronic lung disease (*n*, %)	0	1 (5.5%)	0	NS
Chronic kidney disease (*n*, %)	0	0	2 (11.7%)	NS
Diabetes (*n*, %)	0	2 (11.1%)	6 (35.3%)	NS
Days elapsed between the onset of symptoms and admission	—	6.7 ± 3.6	5.4 ± 4.1	NS

*Note:* Data are presented as mean ± standard deviation, as median (minimum, maximum) or numbers and percentages. Comparison of moderate infections versus severe infections on the day of admission versus healthy controls. Differences were considered significant when the *p*‐value was equal to or less than 0.05.

Abbreviations: BMI, body mass index; HC, healthy control; NA, data not available; NS, not significant.

As detailed in Table 1, patients with severe infection were significantly older and had a higher prevalence of hypertension compared to the moderate infection group, though the gender distribution was comparable. For both groups, symptoms (predominantly fever, cough, and dyspnea) typically began about 1 week before admission. Of the 35 total patients, 24 recovered and were discharged. However, 11 patients died during hospitalization, all from the severe cohort. The median age of the deceased was 67.6 years, and five were females aged 45 or older. Additionally, patients with severe COVID‐19 had significantly longer hospital stays. For comparative analysis, 14 healthy individuals served as controls. These individuals were uninfected, unvaccinated, non‐hospitalized, and symptom‐free during the pandemic. Their control status was confirmed by negative results for anti‐SARS‐CoV‐2 spike and nucleocapsid antibodies (analyzed using the Roche Cobas system).

The laboratory and clinical characteristics of the patients with moderate and severe infections are presented in Table 2.

**Table 2:** Laboratory and clinical characteristics of patients.

Laboratory parameters	Patients with moderate infection (*n* = 18)	Patients with severe infection (*n* = 17)	*p*‐Value
Lymphocyte count (cells/mm^3^)	0.8 ± 0.17	0.66 ± 0.21	<0.01
LDH (U/L)	431 ± 232	926 ± 476	<0.001
D‐dimer (ng/mL)	1027 (228, 24,582)	1597 (681, 329,800)	NS
Ferritin (ng/mL)	1035 (42, 6155)	1117 (32, 9667)	NS
CRP (mg/dL)	9.6 ± 6.7	12.8 ± 7.7	NS
IL‐6 (pg/mL)	62 (7, 143)	61 (31, 924)	NS
Clinical parameters			
Days of hospitalization	8 ± 5	15 ± 12	<0.05
Mortality (*n*, %)	0	11 (64.7%)	—
SOFA score	2 (0.4)	3 (0, 9)	<0.05
SAPS	—	33 (13, 71)	—
CT score	11 ± 4.8	15 ± 6.8	<0.05
PaO_2_/FiO_2_ on admission	194 (32, 410)	67 (43, 388)	<0.03
Mechanical ventilation (days)	—	4 (0, 19)	—
Kidney replacement therapy (*n*, %)	—	3 (17%)	—
Vasopressor therapy (*n*, %)	—	13 (76%)	—
White blood cell count (G/L)	7.48 ± 3.29	10.49 ± 5.58	NS

*Note:* Data are presented as mean ± standard deviation or as median (minimum, maximum). Differences were considered significant when the *p*‐value was equal to or less than 0.05. PaO_2_: partial pressure of oxygen; FiO_2_: fraction of inspired oxygen. For statistical comparisons, independent‐samples *t*‐test and Mann–Whitney *U* tests were performed following the normality test.

Abbreviations: CRP, C‐reactive protein; CT score, compute tomography score; IL‐6, interleukin‐6; LDH, lactate dehydrogenase; NS, not significant; SAPS, simplified acute physiology score; SOFA, sequential organ failure assessment.

Upon admission, significant clinical differences were observed between the two patient groups. Specifically, lymphocyte counts and LDH levels were significantly different between the severe and moderate infection cohorts. All patients with severe infection presented with acute respiratory distress syndrome and had significantly worse CT scores (reflecting more infected lung lobes) [32]. Furthermore, only the severe COVID‐19 infection group required organ support therapies, including mechanical ventilation, kidney replacement, and vasopressor therapy, leading to significantly longer hospital stays compared to those of the patients with moderate COVID‐19.

Peripheral venous blood samples were collected from all patients upon admission before any pharmaceutical treatment was initiated using standard closed‐system tubes. Written informed consent was secured from all participants or their legal representatives. Treatment was managed independently according to the local hospital protocol and was not influenced by the study.

### 2.2. Lymphocyte Separation, Cryopreservation, and Thawing

Peripheral blood mononuclear cells (PBMCs) were initially separated from heparinized venous blood using a Ficoll‐Paque density gradient (GE Healthcare, USA). The PBMC fraction was washed in complete RPMI 1640 medium (Lonza, Switzerland) supplemented with 10% fetal calf serum (FCS). For cryopreservation, cells were counted, centrifuged, and resuspended in inactivated human AB serum containing 10% DMSO (Sigma–Aldrich, USA) as a cryoprotectant. The preserved cells were stored long‐term in a mechanical freezer at −80°C. Before examinations, samples were thawed in a 37°C water bath, resuspended in RPMI 1640 medium, and washed twice to completely remove the residual DMSO content. Cell viability was assessed utilizing trypan blue exclusion prior to flow cytometric analysis. Setting a mandatory threshold (e.g., >90 viability) where any sample falling below the line is excluded from the dataset.

### 2.3. Flow Cytometry

Before surface staining, Fc receptor‐expressing monocytes were blocked for 10 min using the Human TruStain FcX Blocking Solution (BioLegend, USA). For flow cytometric labeling, 10^6^ thawed PBMCs were incubated with a combination of fluorochrome‐conjugated monoclonal antibodies (Supporting Information 1: Table S1) for 30 min at room temperature in the dark. Following a wash step, cells were resuspended in 300 μL PBS (BioSera, France) containing 1% paraformaldehyde (PFA) and stored at 4°C in the dark until FACS analysis. In addition to cell quantification, the relative expression levels of various surface markers were measured using mean fluorescence intensity (MFI). Measurements were performed using a BD FACS Canto II flow cytometer (BD Immunocytometry Systems, Belgium), with data acquisition managed by BD FACS Diva V6 software (BD Biosciences, USA). The spectral overlap was corrected using an automated compensation matrix calculated in BD FACSDiva. Single‐stain compensation controls were prepared using BD CompBeads Anti‐Mouse Ig for each fluorophore utilized in the panel. Gating thresholds and matrix accuracy were visually verified using fluorescence minus one (FMO) controls. The differentiation of the CD56dim and CD56bright subsets was based on the density of the CD56 surface marker. Data analysis was conducted using FCS Express V4 software (De Novo Software, USA).

### 2.4. Intracellular Staining

After surface labeling, cells were washed, fixed using 4% PFA for 10 min, and then washed again with PBS. To permeabilize the cells, a 1:10 dilution of FACS Permeabilizing Solution 2 (BD Biosciences, USA) was used for 10 min in complete darkness. Next, samples were washed and incubated with anti‐human granzyme A, granzyme B, and perforin antibodies for 30 min in the dark. Finally, the cells were washed with PBS, fixed using 1% PFA, and stored at 4°C in the dark until FACS analysis.

### 2.5. NK Cell Degranulation Assay

The degranulation potential of the isolated PBMCs was assessed by incubating the cells for 4 h at 37°C with FITC‐conjugated anti‐human CD107a monoclonal antibody, along with the activators ionomycin and phorbol myristate acetate (PMA) (Sigma–Aldrich, USA). Following activation, samples were washed, resuspended in PBS, and stained with a combination of antibodies for 30 min to specifically identify the CD8+ and CD8− NK cell subpopulations. Finally, cells were washed in PBS, fixed with 1% PFA, and analyzed by FACS.

### 2.6. Statistical Analyses

Statistical analyses were conducted in R version 4.2.2 (R Development Core Team, 2022). Prior to analysis, all response variables except ratio variables were natural log‐transformed to improve compliance with model assumptions. The suitability of transformations and model assumptions was assessed by visual inspection of diagnostic plots, including quantile–quantile (Q–Q) plots and residual‐versus‐fitted plots, to evaluate residual normality and homogeneity of variances.

One‐way ANOVA was used to test for differences among patient groups for all measured response variables. Two‐way ANOVA was used to evaluate the effects of the patient group and marker expression (CD8), including their interaction terms, on the respective response variables. Pairwise comparisons among groups were performed using Tukey’s honestly significant difference (HSD) post hoc tests, which account for multiple comparisons by controlling the family‐wise error rate. Statistical significance was assessed at *α* = 0.05 [33]. Given that ICU patients were significantly older than those in the other groups, we analyzed our data by including age as an additive covariate to enhance the accuracy of our statistical evaluation.

## 3. Results

### 3.1. Ratio of the CD8‐Positive and Negative NK‐Cell Subpopulation in COVID‐19 Patients and Healthy Controls

**Figure 1:**
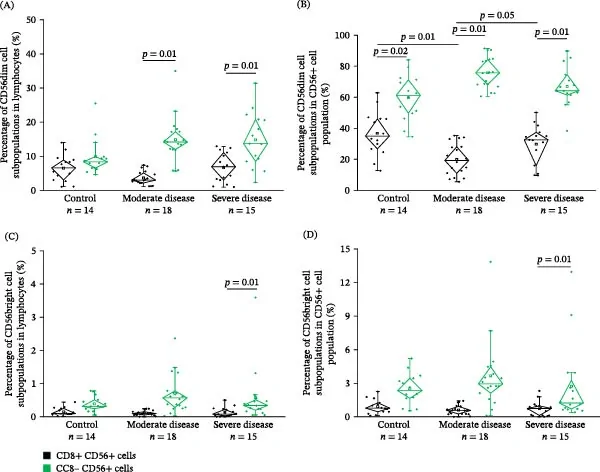
The percentage of CD56+ cell subsets in COVID‐19 patients with moderate or severe symptoms, and in healthy controls. The determined percentages of the CD8+ and CD8− CD56+ cell subsets in the lymphocyte gate (A, C) and in the CD56+ cell gate (B, D) in the peripheral blood of COVID‐19 patients with moderate or severe symptoms and healthy controls. The solid bars represent the medians of the measured samples for each group (control: 14, moderate disease: 18, severe disease: 15), respectively. The boxes indicate the interquartile ranges, the error bars represent the variability of the minimum, maximum, and any outlier data points in comparison to the interquartile range. The middle square within the box represents the mean value. Statistical significance was assessed by two‐way ANOVA, and differences with *p* ≤ 0.05 were considered statistically significant and are indicated in the figure.

To characterize the NK cell subsets, multicolor flow cytometric analyses were applied (Supporting Information 2: Figure S1). Comparing the ratio of the CD56dim subsets in the lymphogate, the CD8− counterpart was significantly higher than the CD8+ cell subset only in the COVID‐19 patients groups (Figure 1A). Furthermore, when we performed this comparison in the gated NK‐cell population, the ratio of the CD8− CD56dim subset was significantly higher than that of the CD8+ subset in all investigated groups (Figure 1B). Moreover, in patients with moderate symptoms, the CD8+ subset ratio was significantly lower than that in the severe group and the healthy controls (Figure 1B).

In the CD56bright subpopulation, a significantly higher ratio of the CD8− subset was detected compared to the CD8+ counterpart but only in patients diagnosed with severe COVID‐19 (Figure 1C,D).

### 3.2. IC Receptor Expression by the CD8‐Positive and Negative NK‐Cell Subpopulation in COVID‐19 Patients and Healthy Controls

**Figure 2:**
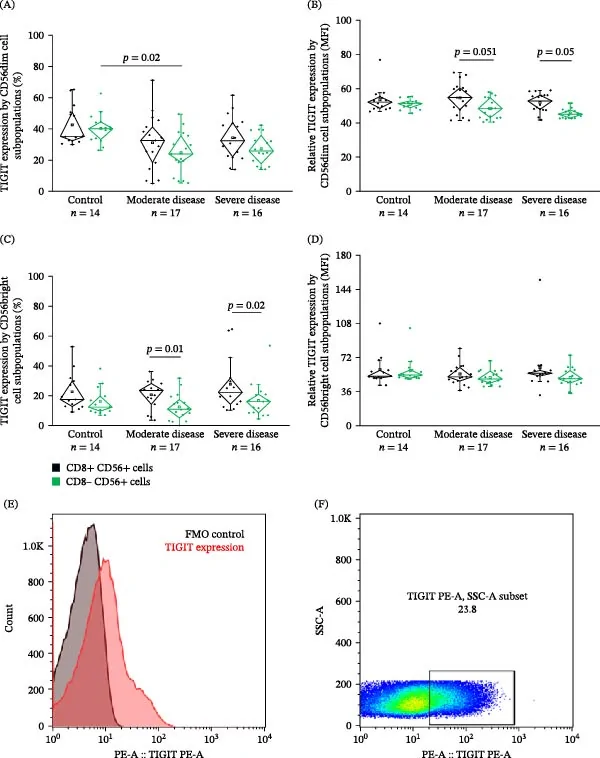
TIGIT expression by CD56dim and CD56bright subpopulations in COVID‐19 patients with moderate or severe symptoms, and in healthy controls. The surface expression levels (A, C) and the MFI (B, D) of the TIGIT receptor on CD8+ and CD8− CD56+ cells, comparing COVID‐19 patients with moderate or severe symptoms and healthy controls. The solid bars represent medians of the measured samples in each group (control: 14, moderate disease: 17, severe disease: 16), respectively. The boxes indicate the interquartile ranges, the error bars represent the variability of the minimum, maximum, and any outlier data points in comparison to the interquartile range. The middle square within the box represents the mean value. Statistical significance was assessed by two‐way ANOVA, and differences with *p* ≤ 0.05 were considered statistically significant and are indicated in the figure. To assess TIGIT receptor positivity, a fluorescent minus one (FMO) control was used (E). Representative FACS plot shows the expression of TIGIT surface marker (F).

Investigating the inhibitory TIGIT molecule, the receptor expression was significantly decreased in the moderate symptoms group CD8− CD56dim cells comparing to the control group CD8− counterpart (Figure 2A). The MFI, which represents relative TIGIT expression, was significantly lower in CD8− CD56dim cells than in CD8+ counterparts but only in the two COVID‐19 patient groups (Figure 2B). In the CD56bright subpopulation, significantly lower receptor expression was observed in the CD8− subset compared to the CD8+ subset in both COVID‐19 patient groups (Figure 2C). However, no significant differences in the TIGIT MFI were observed in CD56bright cells (Figure 2D).

**Figure 3:**
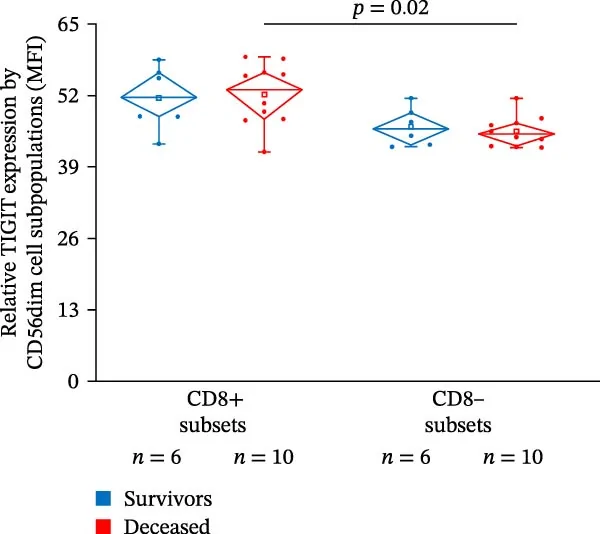
Relative TIGIT expression on CD56dim cell subpopulations within the CD8+ and CD8− subsets, comparing data from survivors and deceased patients derived from severe disease group. The MFI of TIGIT expression on CD56+dim cell subpopulations within the CD8+ and CD8− subsets, comparing data from survivors and deceased patients derived from patients with severe disease. The solid bars represent medians of the measured samples in the severe disease group (survived: 6, deceased: 10), respectively. The boxes indicate the interquartile ranges, the error bars represent the variability of the minimum, maximum, and any outlier data points in comparison to the interquartile range. The middle square within the box represents the mean value. Statistical significance was assessed by two‐way ANOVA, and differences with *p* ≤ 0.05 were considered statistically significant and are indicated in the figure.

TIGIT MF was significantly decreased in the CD8−CD56dim cells from deceased patients compared with their CD8+ counterparts (Figure 3).

**Figure 4:**
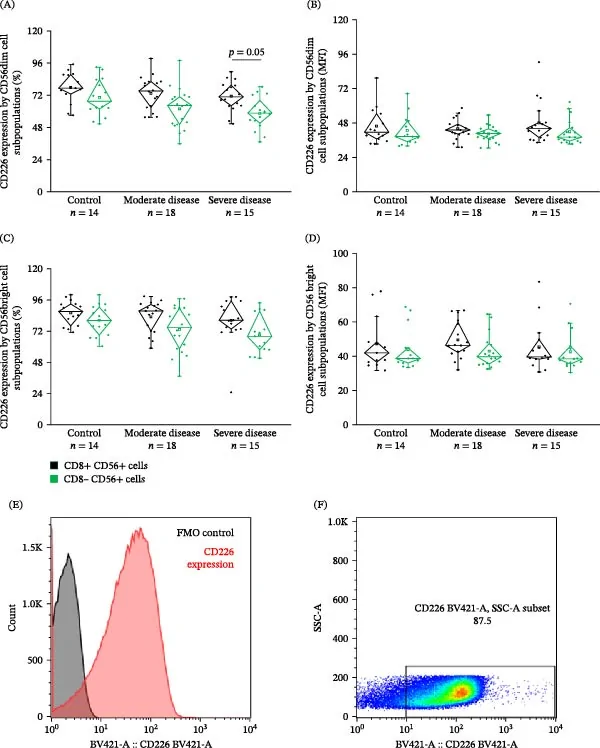
CD226 expression by CD56dim and CD56bright subpopulations in COVID‐19 patients with moderate or severe symptoms and in healthy controls. The surface expression levels (A, C) and the MFI (B, D) of the CD226 activatory receptor on CD8+ and CD8− CD56+ cells, comparing COVID‐19 patients with moderate or severe symptoms and healthy controls. The solid bars represent the medians of the measured samples in each group (control: 14, moderate disease: 18, severe disease: 15), respectively. The boxes indicate the interquartile ranges, the error bars represent the variability of the minimum, maximum, and any outlier data points in comparison to the interquartile range. The middle square within the box represents the mean value. Statistical significance was assessed by two‐way ANOVA, and differences with *p* ≤ 0.05 were considered statistically significant and are indicated in the figure. To assess CD226 receptor positivity, an FMO control was used (E). Representative FACS plot shows the expression of CD226 surface marker (F).

Analyzing the activatory counterpart of TIGIT, the CD226 expression was assessed across NK‐cell subsets. Surface expression of CD226 was significantly lower in the CD8− CD56dim subset compared to the CD8+ CD56dim cells, but this difference was observed only in patients with severe disease (Figure 4A). However, no significant differences were detected in the CD226 MFI (Figure 4B). In the CD56bright subpopulation, neither surface expression nor MFI of CD226 differed significantly between CD8− and CD8+ cells (Figure 4C,D).

**Figure 5:**
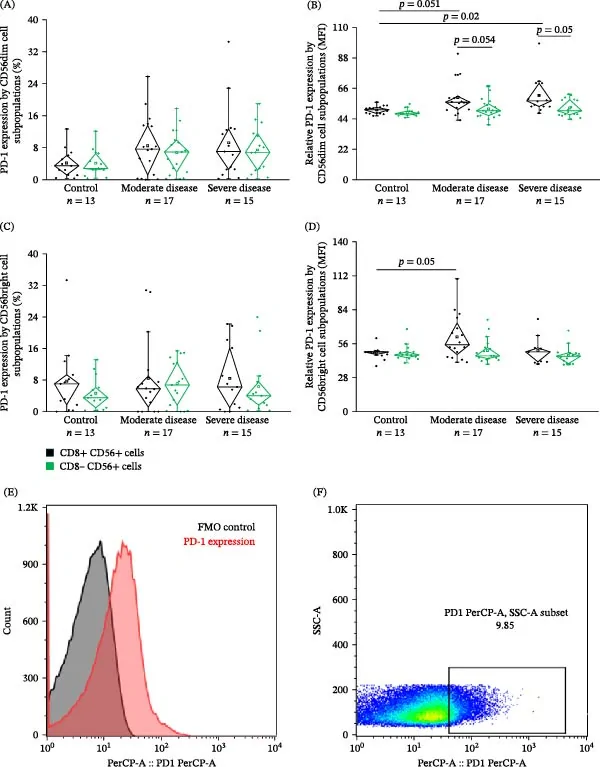
PD‐1 expression by CD56dim and CD56bright subpopulations in COVID‐19 patients with moderate or severe symptoms, and in healthy controls. The surface expression levels (A, C) and the MFI (B, D) of the PD‐1 receptor on CD8+ and CD8− CD56+ cell subsets, comparing COVID‐19 patients with moderate or severe symptoms and healthy controls. The solid bars represent medians of the measured samples in each group (control: 13, moderate disease: 17, severe disease: 15), respectively. The boxes indicate the interquartile ranges, the error bars represent the variability of the minimum, maximum, and any outlier data points in comparison to the interquartile range. The middle square within the box represents the mean value. Statistical significance was assessed by two‐way ANOVA, and differences with *p* ≤ 0.05 were considered statistically significant and are indicated in the figure. To determine the positivity of the PD‐1 receptor, FMO control was used (E). Representative FACS plot shows the expression of PD‐1 surface marker (F).

No significant differences in the surface frequency of PD‐1–expressing cells were observed among the investigated groups (Figure 5A). However, the relative expression of this inhibitory receptor, as measured by MFI, was significantly higher in the CD8+ CD56dim subset in both moderate and severe disease groups (Figure 5B). Correspondingly, PD‐1 MFI was significantly reduced in the CD8− CD56dim subsets of patients with moderate and severe symptoms (Figure 5B). Furthermore, a significantly reduced PD‐1 MFI was observed in the CD8− subsets of the moderate and severe patient groups compared with their CD8+ counterparts (Figure 5B).

Within the CD56bright population, no significant differences in PD‐1 surface expression were detected between groups (Figure 5C). Nevertheless, a significantly increased relative PD‐1 expression was observed in the CD8+ CD56bright subset of patients with moderate symptoms compared to that in healthy controls (Figure 5D).

**Figure 6:**
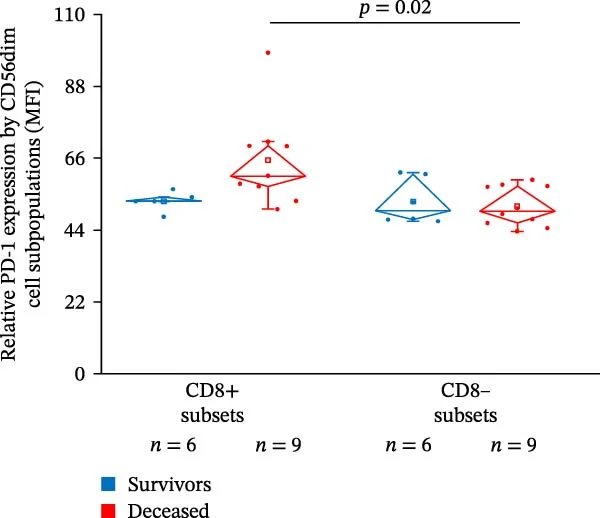
Relative PD‐1 expression on CD56dim cell subpopulations within the CD8+ and CD8− subsets, comparing data from survivors and deceased patients derived from severe disease group. The MFI of PD‐1 expression on CD56+dim cell subpopulations within the CD8+ and CD8− subsets, comparing data from survivors and deceased patients derived from patients with severe disease. The solid bars represent medians of the measured samples in the severe disease group (survived: 6, deceased: 9), respectively. The boxes indicate the interquartile ranges, the error bars represent the variability of the minimum, maximum, and any outlier data points in comparison to the interquartile range. The middle square within the box represents the mean value. Statistical significance was assessed by two‐way ANOVA, and differences with *p* ≤ 0.05 were considered statistically significant and are indicated in the figure.

In the severe disease group, this difference appears to be primarily driven by a marked reduction in PD‐1 MFI in CD8− CD56dim cells compared to their CD8+ counterparts in deceased patients (Figure 6).

### 3.3. Degranulation Activity of the CD8‐Positive and Negative NK‐Cell Subpopulation in COVID‐19 Patients and Healthy Controls

**Figure 7:**
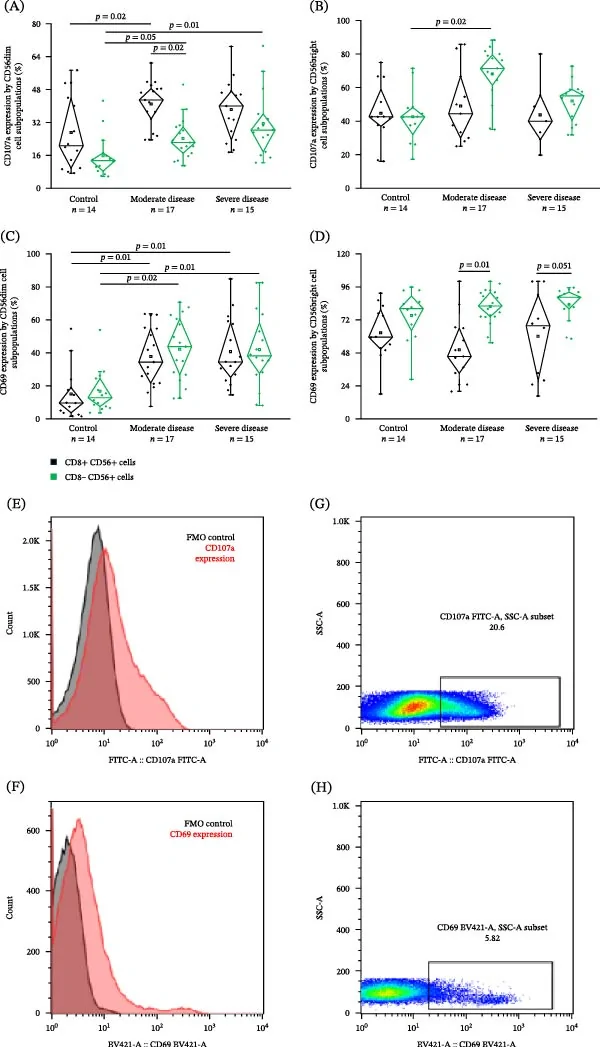
CD107a and CD69 expressions by CD56dim and CD56bright subpopulations in COVID‐19 patients with moderate or severe symptoms, and in healthy controls. The CD107a expression (A, B) and the CD69 expression (C, D) on CD8+ and CD8− CD56+ cells, comparing COVID‐19 patients with moderate or severe symptoms and healthy controls. The solid bars represent medians of the measured samples in each group (control: 14, moderate disease: 17, severe disease: 15), respectively. The boxes indicate the interquartile ranges, the error bars represent the variability of the minimum, maximum, and any outlier data points in comparison to the interquartile range. The middle square within the box represents the mean value. Statistical significance was assessed by two‐way ANOVA, and differences with *p* ≤ 0.05 were considered statistically significant and are indicated in the figure. To determine the positivity of CD107a‐ (E) and CD69 (F) molecules, FMO control was used (E). Representative FACS plots show the expression of CD107a (G) and CD69 (H) markers.

Following the degranulation assay, CD56dim cells exhibited significantly increased CD107a expression in patients with moderate disease, as well as in the CD8− subset of patients with severe COVID‐19, compared to healthy controls (Figure 7A).

In the CD56bright population, degranulation activity (assessed by CD107a expression) was significantly elevated in the CD8− subset of patients with moderate symptoms compared to CD8− CD56bright cells from healthy individuals (Figure 7B).

Regarding early activation, CD69 expression was significantly increased in both CD56dim subsets of COVID‐19 patients compared to that in healthy controls (Figure 7C). In contrast, within the CD56bright population, CD69 expression was significantly higher in the CD8− subset compared to the CD8+ subset in both COVID‐19 patient groups (Figure 7D).

**Figure 8:**
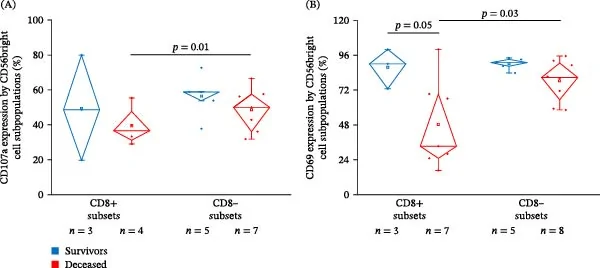
CD107a (A) and CD69 (B) expression on CD56bright cell subpopulations within the CD8+ and CD8− subsets, comparing data from survivors and deceased patients derived from severe disease group. Expression of CD107a and CD69 markers on CD56bright cell subpopulations within the CD8+ and CD8− subsets, comparing data from survivors and deceased patients derived from patients with severe disease. The solid bars represent medians of the measured samples in the severe disease group (survived: 3 and 5, deceased: 4, 7, and 8), respectively. The boxes, indicate the interquartile ranges, the error bars represent the variability of the minimum, maximum, and any outlier data points in comparison to the interquartile range. The middle square within the box represents the mean value. Statistical significance was assessed by two‐way ANOVA, and differences with *p* ≤ 0.05 were considered statistically significant and are indicated in the figure.

No significant difference was observed in the overall severe disease group; subgroup analysis revealed that CD8− CD56bright cells from deceased patients displayed significantly higher CD107a expression compared to their CD8+ counterparts (Figure 8A). Furthermore, CD69 expression was significantly reduced in CD8+ CD56bright cells from deceased patients compared with that of survivors (Figure 8B). Notably, in the deceased group, CD69 expression remained significantly higher in the CD8−subset than in the CD8+ subset (Figure 8B).

### 3.4. Intracellular Cytotoxic Molecule Content in CD8‐Positive and Negative NK‐Cell Subpopulations in COVID‐19 Patients and Healthy Controls

**Figure 9:**
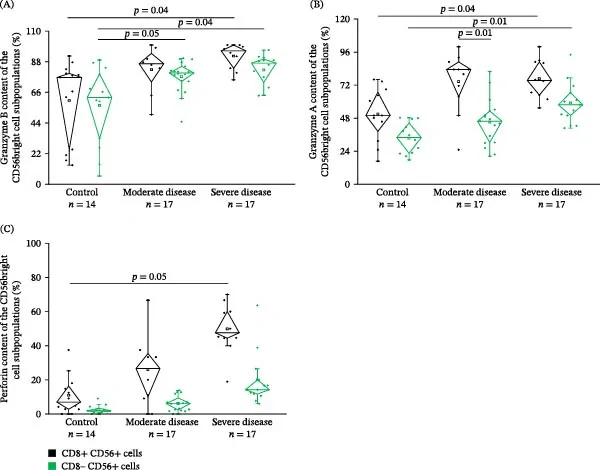
Intracellular granzyme A, granzyme B, and perforin content of the CD56bright subpopulations in COVID‐19 patients with moderate or severe symptoms, and in healthy controls. The intracellular content of granzyme B (A), granzyme A (B), and perforin (C) molecules within the CD8+ and CD8− CD56bright cells comparing COVID‐19 patients with moderate or severe symptoms and healthy controls. The solid bars represent medians of the measured samples in each group (control: 14, moderate disease: 17, severe disease: 17), respectively. The boxes indicate the interquartile ranges, the error bars represent the variability of the minimum, maximum, and any outlier data points in comparison to the interquartile range. The middle square within the box represents the mean value. Statistical significance was assessed by two‐way ANOVA, and differences with *p* ≤ 0.05 were considered statistically significant and are indicated in the figure.

In contrast, the granzyme B and granzyme A contents of the CD8+ and CD8− CD56bright cells were significantly higher but only in the severely infected group compared to the control group (Figure 9A,B). We further observed a significant increase in the CD8− CD56bright cell granzyme B content in the moderate group compared to the controls (Figure 9A). In the case of granzyme A expression, a significantly decreased value was observed in CD8− CD56bright cells compared to the CD8+ counterpart, only in the moderate patient group (Figure 9B). The intracellular perforin content was significantly higher in the CD8+ CD56bright subset in the severe disease group compared to that in the control group (Figure 9C).

**Figure 10:**
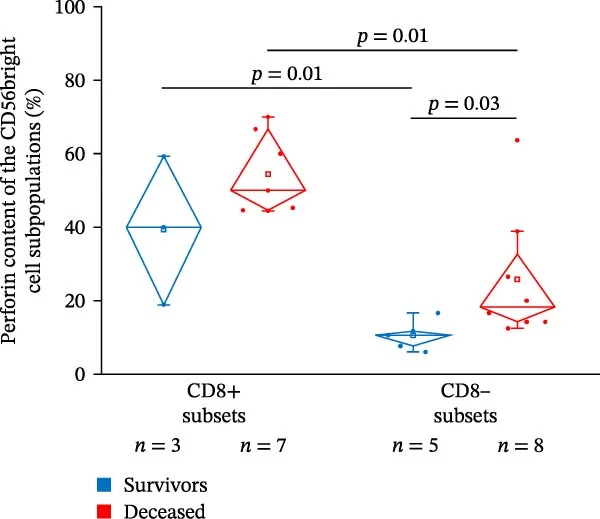
Perforin content of CD56bright cell subpopulations within the CD8+ and CD8− subsets, comparing data from survivors and deceased patients derived from the severe disease group. The perforin content of the CD56bright cell subpopulations within the CD8+ and CD8− subsets, comparing data from survivors and deceased patients derived from patients with severe disease. The solid bars represent the medians of the measured samples in the severe disease group (survived: 3 and 5, deceased: 7 and 8), respectively. The boxes indicate the interquartile ranges, the error bars represent the variability of the minimum, maximum, and any outlier data points in comparison to the interquartile range. The middle square within the box represents the mean value. Statistical significance was assessed by two‐way ANOVA, and differences with *p* ≤ 0.05 were considered statistically significant and are indicated in the figure.

Furthermore, if we split the severe disease group to survived and deceased patients, we observed a significantly decreased perforin expression by the CD8− subsets compared to the CD8+ subset in both groups (Figure 10). Moreover, the CD8− CD56bright cell perforin expression was significantly higher in the deceased groups compared to the CD8+ counterpart (Figure 10.

### 3.5. Intracellular Cytotoxic Molecule Content of the CD107a+ and CD69+ NK‐Cell Subpopulation in COVID‐19 Patients and Healthy Controls

**Figure 11:**
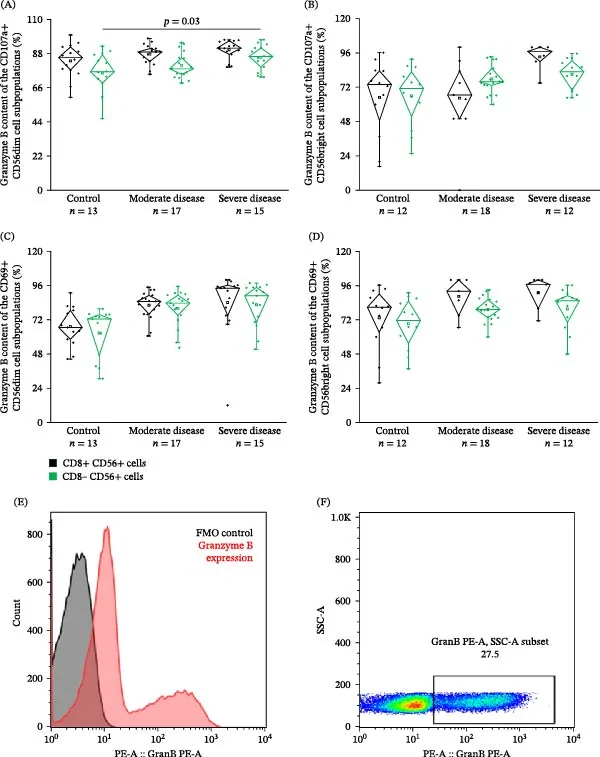
Intracellular granzyme B content of CD56dim and CD56bright subpopulations expressing CD107a or CD69 in COVID‐19 patients with moderate or severe symptoms, and in healthy controls. The intracellular content of granzyme B molecules within the CD8+ and CD8− CD56dim and CD56bright cell subpopulations expressing CD107a (A, B) or CD69 marker (C, D), comparing COVID‐19 patients with moderate or severe symptoms and healthy controls. The solid bars represent medians of the measured samples in each group (control: 12 and 13, moderate disease: 17 and 18, severe disease: 12 and 15), respectively. The boxes indicate the interquartile ranges, the error bars represent the variability of the minimum, maximum, and any outlier data points in comparison to the interquartile range. The middle square within the box represents the mean value. Statistical significance was assessed by two‐way ANOVA, and differences with *p* ≤ 0.05 were considered statistically significant and are indicated in the figure. To determine the positivity of the granzyme B molecule, FMO control was used (E). Representative FACS plot shows the expression of granzyme B intracellular marker (F).

Significantly elevated granzyme B content was observed in the CD107a+ CD8− CD56dim subpopulation in patients with severe disease compared to healthy controls (Figure 11A), while in the case of the CD56bright cells, no statistical difference was measured (Figure 11B). Analysis of CD69+ CD56dim and CD56bright subpopulations revealed no statistically significant differences in the granzyme B content (Figure 11C,D).

**Figure 12:**
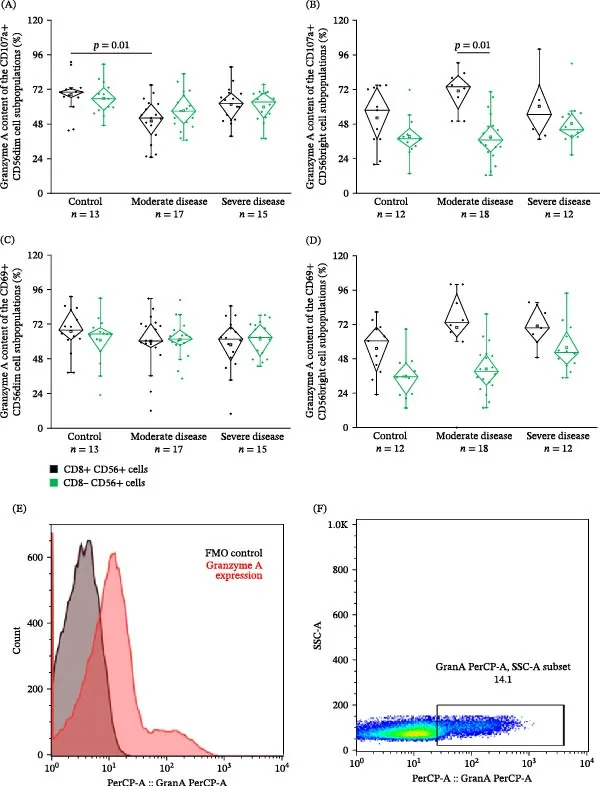
Intracellular granzyme A content of CD56dim and CD56bright subpopulations expressing CD107a (A, B) or CD69 (C, D) in COVID‐19 patients with moderate or severe symptoms, and in healthy controls. The intracellular content of granzyme A molecules within the CD8+ and CD8− CD56dim and CD56bright cell subpopulations expressing CD107a or CD69 marker comparing COVID‐19 patients with moderate or severe symptoms and healthy controls. The solid bars represent medians of the measured samples in each group (control: 12 and 13, moderate disease: 17 and 18, severe disease: 12 and 15), respectively. The boxes indicate the interquartile ranges, the error bars represent the variability of the minimum, maximum, and any outlier data points in comparison to the interquartile range. The middle square within the box represents the mean value. Statistical significance was assessed by two‐way ANOVA, and differences with *p* ≤ 0.05 were considered statistically significant and are indicated in the figure. To determine the positivity of the granzyme A molecule, FMO control was used (E). Representative FACS plot shows the expression of granzyme A intracellular marker (F).

A significantly lower granzyme A content was detected in CD107+ CD8+ CD56dim cells from patients with moderate infection compared to the control group (Figure 12A). Analyzing the CD107a+ CD8− CD56bright cells, the granzyme A content was significantly lower compared to their CD8+ counterparts in patients with moderate symptoms (Figure 12B). No significant differences were observed in the frequency of CD69+ CD56dim and CD56bright subsets among the three study groups (Figure 12C,D).

**Figure 13:**
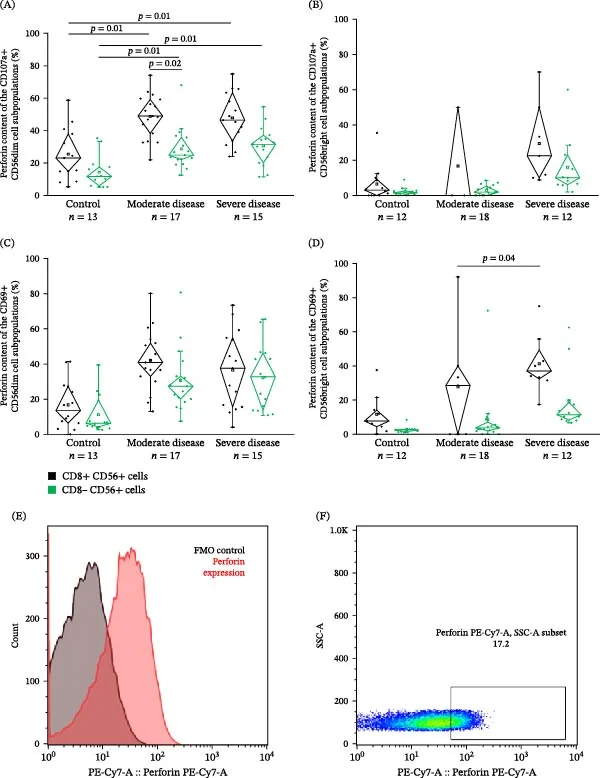
Intracellular perforin content of CD56dim and CD56bright subpopulations expressing CD107a (A, B) or CD69 (C, D) in COVID‐19 patients with moderate or severe symptoms, and in healthy controls. The intracellular content of perforin molecules within the CD8+ and CD8− CD56dim and CD56bright cell subpopulations expressing CD107a or CD69 marker, comparing COVID‐19 patients with moderate or severe symptoms and healthy controls. The solid bars represent medians of the measured samples in each group (control: 12 and 13, moderate disease: 17 and 18, severe disease: 12 and 15), respectively. The boxes indicate the interquartile ranges, the error bars represent the variability of the minimum, maximum, and any outlier data points in comparison to the interquartile range. The middle square within the box represents the mean value. Statistical significance was assessed by two‐way ANOVA, and differences with *p* ≤ 0.05 were considered statistically significant and are indicated in the figure. To determine the positivity of the perforin molecule, FMO control was used (E). Representative FACS plot shows the expression of the perforin intracellular marker (F).

Regarding perforin, significantly higher intracellular levels were measured in all CD107a+ CD56dim subsets in both COVID‐19 patient groups compared with those in healthy controls (Figure 13A). Furthermore, significantly decreased perforin content was revealed in the CD107a+ CD8− CD56dim subset compared to the CD8+ counterpart but only in the moderate group (Figure 13A). In the case of the CD56bright subset, no statistical difference was measured (Figure 13B). However, we did not find a difference in the case of the CD56dim cells (Figure 13C); the intracellular perforin expression was significantly higher in CD69+ CD8+ CD56bright cells from patients with severe infection compared to those with moderate disease (Figure 13D).

## 4. Discussion

Effective immune responses are essential for the resolution of COVID‐19; however, excessive immune activation has been implicated in the high mortality observed in severe cases [34, 35]. The transition from an early innate immune response to a coordinated adaptive response represents a critical determinant of clinical outcome. Although early immune activation is generally protective, dysregulated or exaggerated adaptive responses may impair viral clearance and reduce survival. In acute COVID‐19, much of the observed tissue damage is thought to result from lymphocyte hyperreactivity [4].

To counterbalance immune‐mediated pathology, the upregulation of inhibitory pathways, such as IC molecules, including TIM‐3 and PD‐1, may play a protective role.

Increased expression of these checkpoint receptors in COVID‐19 has been associated with either T‐cell exhaustion or sustained hyperactivation [36, 37]. Nevertheless, the precise immunological predictors that determine the progression to severe disease remain incompletely understood.

Previous studies have identified a reduction in peripheral T‐cell numbers as one of the most common immunological findings in COVID‐19 patients [4, 38]. However, the underlying mechanisms driving this lymphopenia remain incompletely understood [39]. Current evidence is inconsistent regarding whether lymphopenia affects multiple T‐cell populations, such as CD4+ T, CD8+ T, B, and NK cells, or exclusively CD8+ cytotoxic T cells [38, 40]. Moreover, it remains unclear to what extent lymphopenia differs between severe and mild forms of the disease and which specific immune cell populations are most affected.

Jiang et al. [41] reported reduced numbers of CD8+ T cells and NK cells in patients with severe COVID‐19, accompanied by increased functional activity in the remaining cells. In our previous study, no significant differences were observed in the overall frequency of NK‐cell subpopulations [29]. However, a significantly increased frequency of the CD8− CD56dim subset was detected. As this difference was observed across all study groups, it is unlikely to be specifically associated with COVID‐19. However, the reduced proportion of the CD8− CD56dim subset in the moderate group may reflect underlying immunological dysregulation characteristic of this disease stage.

Recent studies have further emphasized the prognostic relevance of NK‐cell alterations in COVID‐19 [42–44]. Comprehensive immunophenotypic analyses have demonstrated that severe disease and fatal outcomes are associated with profound changes in NK‐cell activation, maturation, and functional competence, suggesting that NK‐cell dysregulation reflects the overall immune status of critically ill patients rather than merely antiviral activity [43, 44]. In particular, reduced numbers of circulating NK cells accompanied by increased expression of activation and inhibitory receptors have been associated with poor clinical outcomes, indicating that persistent NK‐cell activation may coexist with functional impairment during severe disease [43, 44]. Moreover, single‐cell transcriptomic analyses have revealed marked remodeling of NK‐cell subsets in patients with severe COVID‐19, characterized by altered cytotoxicity and IC expression that distinguish survivors from non‐survivors [42]. These observations are consistent with our findings demonstrating enhanced activation and cytotoxic features, along with increased inhibitory checkpoint expression, in specific NK‐cell subsets, particularly among patients with fatal outcomes. Although our observational study does not establish causality, the concordance between our phenotypic data and previous reports supports the concept that alterations in NK‐cell phenotype and function are closely associated with disease severity and clinical outcome in COVID‐19 [42–44].

Elevated expression of inhibitory IC receptors, such as PD‐1, has previously been associated with either cellular activation or functional exhaustion [45, 46]. Notably, the NK‐cell phenotype characterized by increased PD‐1 expression has also been associated with enhanced cytotoxic capacity and elevated expression of NK cell‐associated markers in previous studies [38, 47]. Because our study was observational and did not directly assess the checkpoint receptor function, our findings cannot distinguish between these functional states and should therefore be interpreted as phenotypic associations rather than evidence of a specific regulatory mechanism. In the present study, both PD‐1 and TIGIT exhibited a similar pattern of increased relative expression in CD8+ NK‐cell subsets, particularly within the CD56dim population, whereas the activating receptor CD226 showed comparatively limited changes. Together, these findings suggest differential regulation of inhibitory and activating iIC receptors between CD8+ and CD8− NK‐cell subsets during COVID‐19. The increased PD‐1 and TIGIT expression observed in CD8+ NK cells may reflect an adaptive response associated with their potentially greater degranulation activity, while the elevated CD226 expression detected in CD8+ CD56dim cells from critically ill patients may indicate altered immune homeostasis associated with severe disease. However, further functional and mechanistic studies are required to determine the biological significance of these phenotypic differences.

With respect to markers of degranulation, CD107a expression was significantly increased in both CD56dim subpopulations during coronavirus infection, regardless of disease severity. Comparable findings were observed for the early activation marker CD69, whose expression was significantly increased in CD56dim cells. This elevation may reflect increased activation of these cells during the inflammatory immune response accompanying viral infection. Given that the CD56bright subpopulation predominantly comprises cytokine‐producing cells, we did not observe significant changes in the expression of the previously mentioned molecules compared to those of the control group. However, the increased CD69 expression observed on CD8− CD56bright cells may be associated with the reduced PD‐1 and TIGIT MFI detected in this subset. Together, these findings are consistent with an enhanced activation state and a potentially greater capacity for Th1‐type cytokine production by CD8− CD56bright cells during COVID‐19 infection. Moreover, the increased expression of cytotoxicity‐associated molecules in CD8− CD56bright cells from deceased patients may indicate that this subset is associated with uncontrolled inflammatory immune responses, potentially reflecting a failure of regulatory mechanisms in severe disease.

As previously noted, CD56bright cells are primarily characterized as cytokine‐producing cells; however, they can also produce substantial amounts of cytotoxic molecules [48]. In our study, the production of these molecules was significantly higher in COVID‐19 patients compared to that in healthy controls, which may be consistent with an enhanced antiviral immune response. Furthermore, within the moderate group, significantly higher levels of granzyme A were measured in the CD8+ CD56bright and CD8+ CD107a+ CD56bright cell subpopulations. These findings further emphasize the comparatively greater degranulation potential of this subset relative to the CD8− CD56bright population.

This hypothesis is further supported by the elevated perforin expression detected in the CD8+ CD56bright subpopulation in both survivors and deceased patients. As no significant differences in the perforin content were observed in CD56dim cells across the investigated groups, the elevated perforin levels detected in CD107a+ cells may be associated with an enhanced antiviral response. Furthermore, the elevated perforin levels observed in the CD8+ CD56dim subpopulation within the moderate care group are consistent with increased degranulation capacity in patients with more severe disease.

## 5. Limitations

While our study offers valuable insights, it bears several limitations. First, the low total number of enrolled patients could limit the generalizability of our findings. Second, cryopreservation in −80°C is known to affect the surface receptor expression of the NK cells as well as their cellular functionality and cytotoxic activity. Third, functional assays evaluating NK‐cell cytokine production (e.g., IFN‐γ and TNF‐α secretion) or cytotoxic activity were not performed. Therefore, the observed phenotypic alterations cannot be directly linked to changes in NK‐cell effector function or Th1‐associated immune responses.

## Author Contributions

Ildiko Toth and David Sipos collected the samples. Matyas Meggyes and Livia Mezosi performed experiments. David U. Nagy performed data analysis. Matyas Meggyes, Ildiko Toth, and Laszlo Szereday conceived the study and experiments. Matyas Meggyes, Agnes Peterfalvi, and Laszlo Szereday provided guidance in experiment design, writing, and acquired funding for the study.

## Funding

This study was funded by the University of Pecs Medical School Research Grant (Grants PTE‐AOK KA 2024‐09 and KA 2026‐06).

## Disclosure

All authors contributed to the article and approved the submitted version.

## Ethics Statement

The research protocol was approved by the local Ethics Committee aligned with the Medical School, University of Pécs, Pécs, Hungary (Ethical Registration Number 8759‐PTE/2021). The study protocol conforms to the ethical guidelines of the 2013 revised version of the Declaration of Helsinki.

## Conflicts of Interest

The authors declare no conflicts of interest.

## Supporting Information

Additional supporting information can be found online in the Supporting Information section.

## Supporting information


**Supporting Information 1** Table S1: Fluorochrome conjugated monoclonal antibodies used in the study.

**Supporting Information 2** Figure S1: Gating strategy to identify CD8+ and CD8− CD56+ NK cell subpopulations. The gating strategy for identification of the CD8+ and CD8− CD56+ NK cell subpopulations. Following a doublet exclusion (Figure 1A,B), the lymphocyte gate was determined using forward (FSC‐A) and side scatter (SSC‐A) parameters (C). From the lymphocyte gate, CD3−/CD56+ NK cells were selected (D). Based on the density of the CD56 receptor, CD56dim, and CD56bright NK subpopulations were identified (E). Using the CD8 marker within the CD3−/CD56+ quadrant, the histogram illustrates the presence of the CD8 receptor (F). Based on the combination of CD56 and CD8 receptor densities, the four CD56+ NK cell subpopulations were differentiated (G).

## Data Availability

The data that support the findings of this study are available from the corresponding author upon reasonable request.
