# Supplementary material for: Phenotypic Alterations of CD8+ and CD8− CD56+ NK Cell Subsets in Moderate and Severe COVID‐19 Disease
Source: J Immunol Res. 2026 Sep 12;2026:8647361. doi: 10.1155/jimr/8647361 (PMC13570445; doi:10.1155/jimr/8647361)
Supplement: Supplementary file 1 [file JIMR-2026-8647361-s001.docx]

**Supplementary table 1.** Fluorochrome conjugated monoclonal antibodies used in the study.

| **Antigen** | **Format** | **Clone** | **Isotype** | **Company** | **CAT** |
| --- | --- | --- | --- | --- | --- |
| **CD3** | BV510 | UCHT1 | Mouse BALB/c IgGκ | BD Biosciences | 563109 |
| **CD8** | APC-H7 | SK1 | Mouse BALB/c IgG1, κ | BD Biosciences | 560179 |
| **CD56** | APC | B159 | Mouse IgG1, κ | BD Biosciences | 555518 |
| **CD69** | BV421 | FN50 | Mouse IgG1, κ | BD Biosciences | 562884 |
| **CD107a** | FITC | H4A3 | Mouse BALB/c IgG1, κ | BD Biosciences | 555800 |
| **CD226** | BV421 | DX11 | Mouse BALB/c IgG1, κ | BD Biosciences | 742493 |
| **Granzyme A** | PerCp Cy5.5 | CB9 | Mouse IgG1, κ | Biolegend | 507216 |
| **Granzyme B** | PE | GB11 | Mouse BALB/c IgG1, κ | BD Biosciences | 561142 |
| **PD-1** | PerCp Cy5.5 | EH12.1 | Mouse IgG1, κ | BD Biosciences | 561273 |
| **Perforin** | PE-Cy7 | dG9 | Mouse IgG2b, κ | Biolegend | 308126 |
| **TIGIT** | PE | A1553G | Mouse IgG2a,  | Biolegend | 372704 |
