# Supplementary material for: Phenotypic Alterations of CD8+ and CD8− CD56+ NK Cell Subsets in Moderate and Severe COVID‐19 Disease
Source: J Immunol Res. 2026 Sep 12;2026:8647361. doi: 10.1155/jimr/8647361 (PMC13570445; doi:10.1155/jimr/8647361)
Supplement: Supplementary file 2 [file JIMR-2026-8647361-s002.docx]

**Supplementary figure**

**Gating strategy to identify** **CD8+ and CD8- CD56+ NK cell subpopulations.**

The gating strategy for identification of the CD8+ and CD8- CD56+ NK cell subpopulations. Following a doublet exclusion (Fig. 1/A, B), the lymphocyte gate was determined using forward (FSC-A) and side scatter (SSC-A) parameters (C). From the lymphocyte gate, CD3-/CD56+ NK cells were selected (D). Based on the density of the CD56 receptor, CD56dim and CD56bright NK subpopulations were identified (E). Using the CD8 marker within the CD3-/CD56+ quadrant, the histogram illustrates the presence of the CD8 receptor (F). Based on the combination of CD56 and CD8 receptor densities, the four CD56+ NK cell subpopulations were differentiated (G).
